# 4-Methylumbelliferone Treatment and Hyaluronan Inhibition as a Therapeutic Strategy in Inflammation, Autoimmunity, and Cancer

**DOI:** 10.3389/fimmu.2015.00123

**Published:** 2015-03-23

**Authors:** Nadine Nagy, Hedwich F. Kuipers, Adam R. Frymoyer, Heather D. Ishak, Jennifer B. Bollyky, Thomas N. Wight, Paul L. Bollyky

**Affiliations:** ^1^Division of Infectious Diseases and Geographic Medicine, Department of Medicine, Stanford University School of Medicine, Stanford, CA, USA; ^2^Department of Pediatrics, Stanford University School of Medicine, Stanford, CA, USA; ^3^Department of Pediatrics and Systems Medicine, Stanford University School of Medicine, Stanford, CA, USA; ^4^Matrix Biology Program, Benaroya Research Institute, Seattle, WA, USA

**Keywords:** hyaluronan, 4-methylumbelliferone, hymecromone, immune diseases, cancer

## Abstract

Hyaluronan (HA) is a prominent component of the extracellular matrix at many sites of chronic inflammation, including type 1 diabetes (T1D), multiple sclerosis, and numerous malignancies. Recent publications have demonstrated that when HA synthesis is inhibited using 4-methylumbelliferone (4-MU), beneficial effects are observed in several animal models of these diseases. Notably, 4-MU is an already approved drug in Europe and Asia called “hymecromone” where it is used to treat biliary spasm. However, there is uncertainty regarding how 4-MU treatment provides benefit in these animal models and the potential long-term consequences of HA inhibition. Here, we review what is known about how HA contributes to immune dysregulation and tumor progression. Then, we review what is known about 4-MU and hymecromone in terms of mechanism of action, pharmacokinetics, and safety. Finally, we review recent studies detailing the use of 4-MU to treat animal models of cancer and autoimmunity.

There have been an increasing number of studies utilizing 4-methylumbelliferone (4-MU) to inhibit hyaluronan (HA) for either experimental or pre-clinical purposes. These studies are notable because of the central role HA plays in many disease processes, including inflammation and cancer progression, and because of the potential utility in repurposing 4-MU, a drug already used in humans for other indications, to treat these diseases.

This review will first briefly summarize the known contributions of HA to inflammation and cancer progression. Then, it will describe the chemistry and pharmacokinetics of 4-MU, particularly in regards to its inhibition of HA production. Finally, it will examine the available clinical data on the use of 4-MU treatment in humans and summarize the available data on safety and efficacy in animal models.

## Hyaluronan

HA is an extracellular matrix (ECM) glycosaminoglycan (GAG). It has many roles in normal tissue function and development, including providing support and anchorage for cells, facilitating cell–cell signaling, and facilitating cell movement and migration (1–4).

HA is synthesized by three, independently regulated HA synthase (HAS) proteins. These generate predominantly high molecular weight-HA (HMW-HA) of between 2 × 10^5^ and 2 × 10^6^ Da (5). These enzymes lengthen HA by repeatedly adding glucuronic acid and *N*-acetyl-glucosamine to the nascent polysaccharide as it is extruded through the cell membrane into the extracellular space (4). HA in circulation is rapidly degraded while HA bound to proteins and incorporated into tissues such as joints, basement membranes, and the vitreous of the eye is longer lived (5–8).

HA catabolism is mediated by endogenous hyaluronidases, by bacterial hyaluronidases, by mechanical forces, and by oxidative stress (9). This catabolism results in a continuum of different-sized HA polymers, including low molecular weight-HA (LMW-HA; <120 kDa) and, ultimately, in HA oligomers. One important factor in determining the longevity and size of HA are its interactions with HA-binding proteins, called hyaladherins that protect HA from catabolism and turnover. These include TNF-stimulated gene-6 (TSG-6) and inter-α-inhibitor (IαI) (10, 11). Hyaladherins are thought to interact with HA in such a way as to promote the formation of macromolecular complexes that modulate leukocyte adhesion and activation, thus influencing the inflammatory response (3, 4, 10).

The main receptors for HA are CD44 and RHAMM. Upon binding to HA, intracellular signaling pathways are activated; consequently, the receptors participate in a variety of cellular functions including lymphocyte activation and tumor metastasis.

HA levels are greatly elevated in injured tissues, with production increasing by as much as 80-fold (4). Because HA is highly hygroscopic (12), this increased HA production is likely to drive edema at sites of injury. Consistent with this, HA has been implicated in vascular permeability changes (13), leukocyte adhesion and egress (14), and migration (15). HA can be organized into a variety of molecular architectures by forming cross-linked complexes with the above mentioned proteins, and can serve as ligands for leukocytes. Such interactions may trap the leukocytes and prevent eventual destruction of the tissue, as well as trap pro-inflammatory mediators (10). These ECM molecules may initiate a cascade of events that promote inflammation by attracting inflammatory cells and promoting their activation (16).

Along with the amount of HA, the size distribution of local HA polymers varies between healthy and inflamed tissues. Longer polymers of HMW-HA typically predominate in most tissues under steady-state conditions while, shorter, LMW-HA polymers predominate at sites of active inflammation (2, 17, 18). In light of these associations, HA size has been called a natural biosensor for the state of tissue integrity (19).

These changes in the size of HA have functional consequences because of the differential impacts of HA polymers of different sizes on injury responses and homeostasis. HMW-HA, which predominates in healthy tissues, typically inhibits inflammation (20–22). Consistent with this, administration of HMW-HA is anti-inflammatory in lung injury models (23), collagen-induced arthritis (24), and a variety of other *in vivo* model systems (25–29). The generally anti-inflammatory properties of HMW-HA may be mediated, in part, through interactions with the HA receptor CD44 [reviewed in Ref. (18)] and/or through hyaladherins known to bind HA, including TSG-6 and IαI (11, 30, 31).

LMW-HA, conversely, is thought to drive local inflammatory responses by acting as a pro-inflammatory “danger signal” or damage-associated molecular pattern (DAMP) through effects on Toll-like receptor (TLR) signaling (3, 32, 33). LMW-HA promotes the activation and maturation of dendritic cells (DCs) (34), drives the release of pro-inflammatory cytokines such as IL-1β, TNF-α, IL-6, and IL-12 by multiple cell types (35–39), drives chemokine expression and cell trafficking (40, 41), and promotes proliferation (42, 43) and angiogenesis (44). In light of these and other data (45), it seems likely that LMW-HA and HA catabolism contribute to the perpetuation of inflammation in multiple tissues.

### HA in chronic inflammation

Many chronic disease processes associated with unremitting inflammation are associated with prolonged increases in HA, including type 2 diabetes (T2D) (46, 47), liver cirrhosis (48), asthma, and other diseases (49–54). These conditions are typically associated with accumulations of LMW-HA [reviewed in Ref. (18)].

LMW-HA may also promote immune dysregulation at these sites. We have reported that LMW-HA inhibits the function of Foxp3+ regulatory T-cells (Treg) (38), a cell type that plays a major role in suppressing autoimmunity (55). Other TLR agonists are known to have similar effects on Treg (56).

Recently (57), we reported that autoimmune insulitis in autoimmune type 1 diabetes (T1D) was associated with islet-specific deposition of HA. Using human T1D tissue samples from cadaveric organ donors obtained through the Juvenile Diabetes Research Foundation (JDRF) National Pancreatic Organ Donor (nPOD) program, we discovered that HA deposits were present in islets from recent-onset T1D donors but not in non-diabetic controls. These T1D-associated HA deposits were also associated with local alterations in hyaladherins, including reduced levels of intra-islet TSG-6 and IαI and increases in mRNA of versican, a pro-inflammatory hyaladherin (57). We have made similar observations in animal models of autoimmune diabetes, including non-obese diabetic (NOD) mice (58) and DORmO mice. Together with recently published histologic and biochemical analyses by our group and others, of islet ECM in non-diabetic human and murine islets (59–62), these data implicated HA and the islet ECM in the onset of T1D.

Along with insulitis, HA is highly abundant within demyelinated lesions in multiple sclerosis (MS) and in experimental autoimmune encephalomyelitis (EAE) (63). It is produced by local astrocytes (63, 64), and is known to contribute to EAE by promoting the extravasation of leukocytes (65) and inhibiting oligodendrocyte maturation (66, 67). Lymphocyte infiltration into the CNS is known to precede HA production by astrocytes in EAE, suggesting that astrocytes may produce HA in response to inflammatory factors produced by lymphocytes (63, 64, 68, 69).

HA has also been implicated in other autoimmune diseases, including rheumatoid arthritis (70, 71), lupus (72), Sjögren’s syndrome (73), and Hashimoto’s thyroiditis (74). There is further evidence that targeting HA receptors, including CD44, may be beneficial in several animal models of autoimmunity, including the NOD mouse model of autoimmune diabetes and the collagen-induced arthritis model of rheumatoid arthritis (75–77), though these effects may result from effects on lymphocyte trafficking or apoptosis rather than effects on the local ECM milieu.

### HA in cancer

There is extensive communication between the tumor microenvironment and cancer cells (78, 79). This communication is thought to govern critical cellular processes in metastasis, including angiogenesis, proliferation, and stimulation of tissue-degrading proteases (80). Consistent with this, *in vivo* and *in vitro* data from different origins and various malignancy grades revealed a positive correlation between tumor aggressiveness and stromal HA expression (81–83).

Different expression patterns of HASes are seen during tumor progression. Aggressive ovarian and breast cancer cells express high levels of HA synthase 2 (HAS2) and lower levels of HA synthase 3 (HAS3) compared to non-aggressive cancer cells (84, 85). Indeed, HAS expression levels are inversely correlated with breast cancer staging grades and patient survival (86). HAS expression patterns may be somewhat cancer specific; for example, metastatic prostate and colon cancer express higher levels of HAS3 than HAS2. HA synthase 1 (HAS1) on the other hand was expressed only at very low levels in these tumors (87, 88).

HA forms inter- and intra-molecular organizations, creating a viscous milieu well suited for tumor growth and metastasis. This HA-rich tumor matrix provides structural integrity, maintenance of homeostasis, release of growth factors, cytokines, and nutrients essential for proliferation (10). HA plays an important role in cancer in intracellular signaling cascades associated with tumor growth (89), tumor cell adhesion (90), neovascularization (91–93), and metastasis (90). Many of these pro-tumorigenic effects are attributable to HA fragments.

Conversely, HMW-HA was recently implicated in the inhibition of tumor progression (94). Tian et al. found that naked mole-rat fibroblasts secrete HMW-HA, which is over five times larger than human or mouse HA. This HMW-HA accumulates in naked mole-rat tissues. Interestingly, once HMW-HA is removed by either knocking down HAS2 or overexpressing hyaluronidase 2 (HYAL2), the naked mole-rat cells become susceptible to malignant transformation and form tumors.

HA is also known to influence the susceptibility of tumors to chemotherapeutic agents (95). HA-evoked anti-cancer drug resistance may be of a physico-mechanical nature as a dense ECM limits the delivery and distribution of therapeutic agents (96) and enzymatic depletion of HA is being explored as a means to improve drug delivery (97). Indeed, hyaluronidase, an enzyme that degrades HA, has been used in tumor therapy in combination with chemotherapeutic agents for over two decades (98).

Another reported approach to facilitate the delivery of chemotherapeutic agents through HA is to use the large volumetric domain of HA to entrain small chemotherapeutic drugs within the HA matrix. The resultant HA/drug formulation accumulates in the microvasculature of the tumor, forming a microembolism that increases drug retention at the tumor site and allows for active tumor uptake through HA receptors (99). As a result, a Phase II clinical trial of specific HA formulations of three anti-cancer drugs have been undertaken (100).

Taken together, these data suggest that HA may create a permissive environment for tumor growth and metastasis.

## 4-Methylumbelliferone

**Figure 1:**
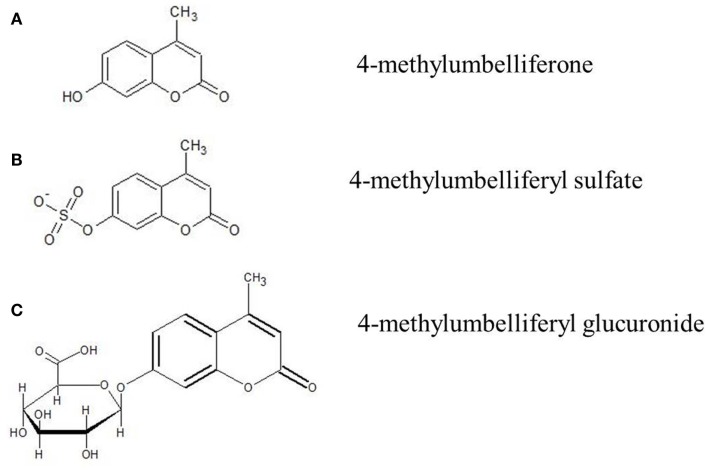
**Molecular structure of 4-MU and its metabolites**. **(A)** 4-Methylumbelliferone (4-MU), **(B)** 4-methylumbelliferyl sulfate (4-MUS), **(C)** 4-methylumbelliferyl glucuronide (4-MUG).

In light of these contributions of HA to inflammation, autoimmunity, and to tumor growth and metastasis, there has been great interest in identifying pharmacologic tools to inhibit HA synthesis. One agent that has received much attention is 4-MU (Figure 1).

4-MU is a derivative of coumarin. Other coumarin derivatives, phenprocoumon (Marcumar^®^) and warfarin (Coumadin^®^), are used in preventive medicine to reduce cardiovascular events due to its anticoagulatory mechanism. Coumarin hydroxylated in position seven is known as umbelliferone and is a natural molecule in plants worldwide. Known representatives of *umbellifera* are lovage (*Levisticum officinale*) and chamomile (*Matricaria recutita*).

4-MU is umbelliferone methylated at position four. It has the IUPAC name 7-hydroxy-4-methylcoumarin and the international free name (INN) hymecromone. It has the molecular formula C_10_H_8_O_3_, a molecular weight of 176.2 kDa, the CAS number is 90-33-5, and a pKa of 7.79. The melting point of 4-MU is 194–195°C. 4-MU is soluble in methanol with heating, DMSO, and in glacial acetic acid. It is slightly soluble in ether or chloroform and practically insoluble in water.

4-MU is known for its fluorescent properties and has an excitation wavelength of 380 nm and an emission wavelength of 454 nm in water. It is colorless at pH 7.0 and exhibits a blue fluorescence at pH 7.5. In light of these properties, it has been used extensively as a pH-sensitive fluorescent indicator in multiple experimental settings.

### 4-MU-mediated inhibition of HA production

The other major experimental use of 4-MU is for HA inhibition. 4-MU has been shown to inhibit HA production in multiple cell lines and tissue types both *in vitro* and *in vivo* (101–108).

**Figure 2:**
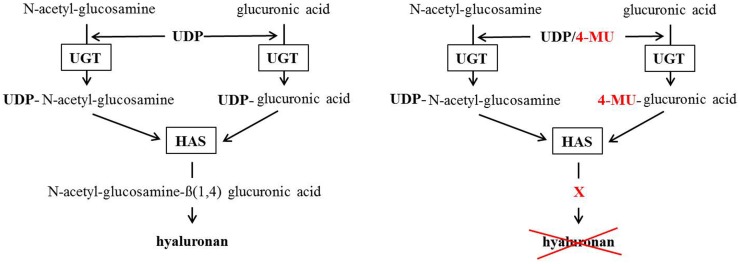
**Postulated 4-methylumbelliferone mechanism of HA synthesis inhibition**. The left scheme shows the normal way HA gets synthesized. The right scheme shows how 4-MU binds to glucuronic acid instead of UDP so the HA-synthases (HAS) cannot build HA.

4-MU is thought to inhibit HA production in at least two ways. First, 4-MU is thought to function as a competitive substrate for UDP-glucuronosyltransferase (UGT), an enzyme involved in HA synthesis (106). HA is produced by the HAS1, HAS2, and HAS3 from the precursors UDP-glucuronic acid (UDP-GlcUA) and UDP-*N*-acetyl-glucosamine (UDP-GlcNAc). These are generated by the transfer of an UDP-residue to *N*-acetylglucosamine and glucuronic acid via the UGT. The availability of UDP-GlcUA and UDP-GlcNAc thereby controls HA synthesis (109). However, when 4-MU is present, it covalently binds through its hydroxyl group at position four to glucuronic acid via the UGT. As a consequence, the concentration of UDP-GlcUA declines in the cytosol and HA synthesis is reduced (Figure 2). 4-MU thereby reduces the UDP-GlcUA content inside the cells and inhibits HA synthesis.

Second, 4-MU reduces expression of HAS mRNA expression (105) as well as mRNA for UDP-glucose pyrophosphorylase and dehydrogenase (110). It is unclear how this second mechanism works or how selective it is for these mRNAs.

### 4-MU effects on other GAGs

4-MU is commonly described as a specific inhibitor of HA synthesis. However, its impact on other GAGs has not been definitively established, to our knowledge.

It was recently reported that 4-MU exerts at least some of its actions via regulation of UDP-glucose dehydrogenase (UGDH), a key enzyme required for both HA and sulfated-glycosaminoglycan (sGAG) production (111). However, other GAGs, such as chondroitin and heparin sulfates, were less sensitive to UDP-GlcUA deficiency. This was suggested to be because they are synthesized in the Golgi apparatus, which has transporters with a very high affinity that pump in UDP sugars from the cytosol that might render inhibition by a competitive substrate such as 4-MU less efficient. In contrast, HA is synthesized at the cytoplasmic membrane.

### 4-MU effects on tumors and cancer cells

The first described use of 4-MU in the context of HA was in 1995 when Nakamura et al. published their study about 4-MU in human skin fibroblasts (112). The postulated 4-MU mechanism was described years later in 2004 by Kakizaki and his group (106).

By far, the greatest experience with 4-MU is in cancer cell lines and *in vivo* models. In 2006, the first *in vivo* study investigating the effect of 4-MU on pancreatic cancer was published (113). More *in vitro* and *in vivo* studies have followed on this subject (114–129). These are detailed in Table 1. The consensus of these studies is that 4-MU inhibits the proliferation, migration, and invasion of multiple cancer cell types, both *in vitro* and *in vivo*.

**Table 1:** **List of experimental studies using 4-MU, broken down by disease type**.

Cancer	Inflammation	Autoimmunity
Pancreatic cancer (113–115)	Non-infectious inflammation (130–132)	CNS autoimmunity (133)
Prostate cancer (134)	Infectious inflammation (135, 136)	Autoimmune arthritis (70)
Skin cancer (107, 116–118)		
Esophageal cancer (129)		
Breast cancer (119–121)		
Liver cancer (122, 123)		
Bone cancer/metastases (126, 128, 137, 138)		
Leukemia (124, 125)		
Ovarian cancer (127)		

Most of these effects are consistent with what is known about the physiologic roles of HA in normal growth and differentiation and how many tumors establish HA-rich matrices to promote their own growth and metastasis. For example, consistent with HA’s role in cell survival pathways, 4-MU treatment is associated with growth arrest and apoptosis of tumor cells (120). Indeed, the apoptotic effect of 4-MU on smooth muscle cells could be rescued with exogenous HA (139). Consistent with the established role of HA in angiogenesis, 4-MU treatment is reported to suppress the new blood vessel growth required for metastases (103, 110).

However, it is not obvious that all of the effects of 4-MU treatment are directly related to HA inhibition. For example, 4-MU was recently reported to inhibit growth of an ovarian tumor cell line via suppression of thymidine phosphorylase (TP) mRNA (127).

In summary, the use of 4-MU to inhibit cancer progression is an active frontier in oncology research, with extensive data in animal models and *in vitro* cell lines supporting further investigation. However, while one can be optimistic about the potential for adjunctive benefit of 4-MU in cancer therapy, much remains unknown and crucial human clinical studies have yet to be done.

### 4-MU effects on inflammation and autoimmunity

There have been more limited investigations into the impact of 4-MU and HA synthesis inhibition in inflammation and autoimmunity. McKallip et al. reported that 4-MU treatment prevented lung injury and reduced inflammatory cytokine levels in mouse models of staphylococcal enterotoxin-mediated (135) and lipopolysaccharide-mediated acute lung injury (136). 4-MU has also been used to inhibit HA production by several human pathogens and their interactions with human cells *in vitro* (140, 141).

4-MU has also been shown to have protective effects on non-infectious inflammation, including renal ischemia and reperfusion (130), and airway inflammation secondary to cigarette smoke (131). 4-MU was shown to restore normoglycemia and promote insulin sensitivity in obese, diabetic mice via increased production of adiponectin (132).

4-MU has also been reported to ameliorate disease in a limited number of mouse models of autoimmune disease. Specifically, 4-MU treatment was beneficial in the collagen-induced arthritis model where it improved disease scores and reduced expression of matrix metalloproteases (MMPs) (70). More recently, 4-MU treatment was demonstrated to prevent and treat disease in the EAE model where it increased populations of regulatory T-cells and polarized T-cell differentiation away from pathogenic, T-helper 1 T-cell subsets and toward non-pathogenic T-helper 2 subsets (133).

These effects point toward a potential role for 4-MU in immune modulation. We have reported that 4-MU treatment prevented cell–cell interactions required for antigen presentation (108) and others have described inhibitory effects on T-cell proliferation (102). These effects are consistent with established roles for HA and its receptors in T-cell proliferation, activation, and differentiation (3, 142, 143). These data also align well with the known effects of 4-MU in lymphoma studies (124, 125).

One pressing question is why 4-MU is anti-inflammatory in multiple systems whereas HA itself has both pro-and anti-inflammatory attributes. One hypothesis is that 4-MU may lead to a preponderance of HMW-HA polymers, with typically anti-inflammatory properties over HA fragments, with typically pro-inflammatory properties. This model assumes that loose HA fragments are more readily cleared than HMW-HA, which is more likely to be integrated into stable matrices and perhaps therefore less subject to rapid turnover. A related notion is that most of the increase in HA production that occurs at times of inflammation is pro-inflammatory in ways that HA produced at times of homeostasis is not. These differences could be mediated either at the level of the different HASes or via cotemporaneous production of hyaladherins. These hypotheses remain to be tested.

There are also indications that 4-MU treatment may make some models of inflammation worse. For example, 4-MU treatment was also associated with worse atherosclerosis in ApoE-deficient mice fed a high-fat diet (104). It is tempting to speculate that HA plays roles in barrier function in some tissues such that its loss leads to enhanced exposure to bacteria or inflammatory mediators.

## Hymecromone

4-MU is already an established therapeutic currently used in humans. Called “hymecromone,” it is used in multiple countries mainly for its choleretic and biliary antispasmodic activity (144–147). Despite being a coumarin derivative, hymecromone does not possess anticoagulant properties. In Europe, hymecromone is an approved drug for use in humans for biliary dyskinesia (original European Union reference date 07/27/1960). For example, in Italy, hymecromone is marketed as a generic named Cantabilin^®^ with a current marketing authorization via the Italian Medicines Agency (AIC no. 02130002) [“Cantabilin^®^ (hymecromone Tablets) (Italian Package Insert)” 2013].

### Clinical experience with hymecromone (4-MU)

The typical approved dosing regimen for adults is 300–800 mg three times/day by mouth (900–2400 mg/day). It is generally available as a tablet with dose strengths of 300–400 mg. Hymecromone is currently not approved for any indication in the U.S., and therefore requires an Investigational New Drug (IND) application from the Food and Drug Administration (FDA) for clinical studies conducted in the U.S.

Several clinical trials in humans, including randomized placebo-controlled, have been published on hymecromone and all demonstrated excellent safety during short-term administration of approved doses (148–154) (Table 2). Taken together, at least 182 patients have been exposed in clinical trials and no serious adverse events from hymecromone were reported. The longest reported duration of administration of hymecromone was a multiple-dose study of oral administration of hymecromone at 1200 mg/day (400 mg three times/day) for 3 month in 20 participants with biliary dyskinesia (152). The tolerability and safety of longer durations of chronic administration is not known, yet this will be necessary to formally establish given the potential need for chronic long-term treatment duration as a therapy in inflammatory or autoimmune conditions.

**Table 2:** **Clinical trials using hymecromone (4-MU) in humans**.

Reference	Patient type	Study type	*n*	Dose	Duration	Adverse events/notes
(153)	Patients requiring cholecystectomy, age >14	Double-blind, randomized, placebo-controlled	25	2400 mg/day × 7.5 days then 1200 mg × 7 days	2 weeks	Decreased drain output and need for post-op analgesics, two pts with mild headaches in treatment group, three with decreased appetite and diarrhea in placebo group
(149)	Post-cholecystectomy dyspepsia, age >60, mean 58.5 years	Double-blind, randomized, placebo-controlled	15	600 mg BID	3 weeks	N/A
(152)	Biliary dyskinesia	Randomized controlled trial vs. tiropramide 300 mg	20	1200 mg daily	3 months	N/A
(149)	Patients requiring cholecystectomy, age 29–84	Placebo-controlled, randomized	13	1600 mg/day	3 weeks	N/A
(155)	Healthy, age 21–35	Pharmacokinetics	8	400 mg IV, 800 mg IV, 600 mg PO solution, 1200 mg PO solution, 1200 mg tablets	Once	N/A
(150)	Healthy, age 22–30	Prospective, double-blind, randomized cross-over study	20	400 mg IV	Once, after meal	N/A
(148)	Healthy	Placebo-controlled, multicenter, randomized	61	600 mg with lunch, 600 mg with dinner	2 weeks	N/A
(154)	Healthy, age 20–37	4-methylumbelliferone PO and IV	20	800 mg × 1 (PO and IV)	Once, with meal	N/A

Overall, the most common side effects during hymecromone treatment are diarrhea or other mild gastrointestinal symptoms [“Cantabilin^®^ (hymecromone tablets) (Italian Package Insert)” 2013]. The diarrhea occurs in 1–10% of patients and appears to be dose-dependent. One study reported a dose of 2400 mg/day (800 mg three times/day) continued for more than 7 days would be expected to result in unacceptable diarrhea. However, this was a study in patients who had undergone bile duct surgery including insertion of a T-drain into the common bile duct. Therefore, whether patients with a normal biliary system would experience the same level of diarrhea at this dose is unknown.

Also of value from a safety perspective is a single dose study in healthy volunteers given as an intravenous dose of 400 and 800 mg (145). Side effects other than those related to intravenous (IV) injection for the 400 mg IV dose included minor dizziness and nausea (four of eight subjects) and “cold sweat” for 5 min (two of eight subjects). After the 800 mg IV dose, side effects reported included “bad after taste” (one of six subjects), nausea and dizziness (three of six subjects), and emesis (two of six subjects). This safety data is of significance as the bioavailability after oral dosing is <3%. Therefore, the systemic exposures after these IV doses were substantially higher than the exposure after typical oral doses. While the safety data at these higher exposures after IV dosing are severely limited given only single dose exposure, it may hint at the tolerability of higher oral doses of hymecromone in humans, which may be necessary for new indications of the drug if higher systemic exposures are required. The overall safety of hymecromone is further supported by animal data noted in the Italian Medicines Agency “package insert” which notes, “acute toxicity has proved to be very low: the LD50, for oral administration is 7593 mg/kg in mice and 6220 mg/kg in rats. Protracted oral administration in the range of 800–2400 mg/kg/day for 3 months and in the rat 400–1000 mg/kg/day for 4 months, has shown excellent tolerability…” [“Cantabilin^®^ (hymecromone tablets) (Italian Package Insert)” 2013]. Contraindications to taking hymecromone include pregnancy and lactation given the lack of safety data in these groups [“Cantabilin^®^ (hymecromone tablets) (Italian Package Insert)” 2013].

Taken together, the clinical experience to date suggests hymecromone is a safe and well-tolerated oral medication. The safety of oral hymecromone doses as high as 2400 mg/day and treatment durations as long as 3 months have been demonstrated in humans and can serve as a benchmark for early stage clinical trials exploring new indications.

### Clinical pharmacology of hymecromone

Hymecromone is extensively metabolized and <1% of a given dose is excreted unchanged in the urine (155, 156). Metabolism of the drug occurs via conjugation to either a glucuronic acid, 4-MUG, or a sulfate (4-MUS) (Figure 1). The glucuronide is the predominant pathway and accounts for over 90% of its metabolism (155, 156). Following conjugation of glucuronic acid to hymecromone, the resulting more hydrophilic metabolite, 4-MUG, is eliminated in the bile and urine (156). Biliary eliminated 4-MUG likely undergoes further enterohepatic recirculation with reabsorption of the metabolite from the intestine and ultimate elimination in the urine via the kidney. This is supported by a healthy volunteer pharmacokinetic study in which 93% of a single intravenous dose of hymecromone was eliminated as the 4-MUG metabolite in the urine (155). However, the precise contribution of enterohepatic recycling in the disposition of hymecromone and its metabolite is not well studied.

Glucuronidation of hymecromone is catalyzed by the UGTs which are a large superfamily of over 20 proteins involved in the Phase II biotransformation of lipophilic xenobiotics and endogenous compounds (157, 158). UGTs are expressed in a wide range of tissues, however, for the purposes of drug biotransformation, the most clinically relevant are located in the liver and intestine (159, 160). Interestingly, hymecromone is a promiscuous molecule in that it is a substrate of most of the major hepatic and intestinal UGTs involved in drug metabolism (158). Consequently, the intestine and liver are very efficient in the metabolism of hymecromone. Pharmacokinetic studies in animals have demonstrated the extraction of hymecromone by the gastrointestinal system (pre-hepatic) to be ~40% and extraction by the liver as high as 97% (156). As a result of this high extraction, the fraction of an administered oral dose of hymecromone that reaches the systemic circulation (post-hepatic) as unchanged drug (i.e., the bioavailability) is very low. In a pharmacokinetic study of hymecromone in healthy volunteers, the systemic bioavailability of hymecromone after oral dosing was <3% (155). As a treatment for biliary colic, the low bioavailability of hymecromone after an oral dose is less of a pharmacokinetic liability. Indeed, the high extraction by the liver may actually be beneficial as the drug is able to concentrate in the hepatic and biliary system.

If first-pass metabolism is bypassed by giving the dose IV, the systemic exposure achieved can be more than 10–30-fold higher than after the same dose given orally (155). However, due to the high clearance of hymecromone, systemic concentrations will decrease rapidly after an IV dose and peripheral exposures will likely be quite low by 4–6 h after a dose (apparent terminal half-life of ~1 h).

The pharmacokinetics of the hymecromone metabolite, 4-MUG, are not well studied. In healthy volunteers, the systemic exposure of 4-MUG after an IV dose was shown to be higher than that of hymecromone (155). Pharmacokinetic data in humans after oral dosing on systemic exposure of 4-MUG are lacking. However, animal data from our group has demonstrated that the median plasma concentration of 4-MUG compared to hymecromone was more than 3,000-fold higher in Balb/C mice on 5% oral hymecromone chow. This animal data highlights the potential importance of understanding 4-MUG pharmacokinetics during oral hymecromone therapy given the expected much higher exposures of the metabolite relative to the parent in peripheral tissues other than the intestine and liver. Future clinical studies of hymecromone in humans would benefit from a more thorough understanding of the pharmacokinetics of the 4-MUG metabolite including whether it is a potentially active moiety.

## The Therapeutic Outlook for Repurposing Hymecromone

The existing *in vitro* and *in vivo* data suggest that hymecromone may have utility as a component of therapeutic regimens directed against HA-producing cancers. There is less data at present to support this strategy in settings of chronic inflammation and autoimmunity but the potential is there as well. However, significant unresolved questions about safety, dosing, and mechanism remain.

While hymecromone has a long and relatively reassuring safety record, many questions remain about its potential repurposing for cancer treatment and other applications. These indications may require much higher dosages than those currently used to treat biliary spasm, introducing the potential for additional side effects. Certainly, the potent effects seen with 4-MU on tumor proliferation, angiogenesis, and migration could have detrimental effects on other tissues. There may also be unanticipated issues related to these novel applications. For example, in mouse models, 4-MU treatment has been linked to a reduced ability to renally excrete electrolytes and fluids (to diuresis) in response to rapid hydration (161). One could envision how this might be problematic if 4-MU were used in conjunction with chemotherapies that are renally cleared.

Long-term hymecromone treatment, rather than the more intermittent use associated with treatment of biliary spasm, might also be associated with unanticipated consequences. For example, we reported that 4-MU treatment was associated with worse atherosclerosis in ApoE-deficient mice fed a high-fat diet (104).

Several clinical pharmacology considerations must also be addressed. The large first-pass metabolism and rapid clearance of hymecromone are obstacles to achieving and maintaining high drug concentrations. This is particularly a concern in peripheral tissues (i.e., pancreas, skin, brain, etc.). Targeting conditions in the intestine and liver will pose less of a problem as these organs likely experience much higher exposures after oral dosing.

Understanding how experimental studies in animal models are likely to relate to human drug dose need and concentration is also likely to be important. Notably, the metabolism and drug disposition of 4-MU in mice may be very different than in humans.

Certainly the successful development of hymecromone will demand robust, pharmacokinetic studies of hymecromone and its metabolites in humans. Such detailed pharmacokinetic understanding will help develop dosing strategies including appropriate dose strength and frequency. These studies will set the stage for evaluation of this promising therapy in human clinical trials.

In summary, there is potential for hymecromone to be developed and repurposed as a safe, long-term adjunctive therapy for cancer treatment or other potential indication. Hymecromone’s long and reassuring clinical track record, its oral route of delivery, and the exciting *in vitro* and *in vivo* data in mice all support further exploration of this therapeutic strategy. However, substantial pharmacologic and safety issues must be addressed in order to facilitate the translation of hymecromone into the clinic.

## Conflict of Interest Statement

The authors declare that the research was conducted in the absence of any commercial or financial relationships that could be construed as a potential conflict of interest.
